# An in vitro study on the risk of non-allergic type-I like hypersensitivity to Momordica charantia

**DOI:** 10.1186/1472-6882-13-284

**Published:** 2013-10-26

**Authors:** Rahsan Ilikci Sagkan

**Affiliations:** 1 Transplantation Laboratory, Division of Immunology and Allergy Diseases, Gulhane Military Medical Academy, Ankara, Turkey

**Keywords:** *Momordica charantia*, Allergy, Basophil activation

## Abstract

**Background:**

*Momordica charantia* (MC) is a tropical plant that is extensively used in folk medicine. However, the knowledge about side effects of this plant is relatively little according to knowledge about its therapeutic effects. The aim of this study is to reveal the effects of non-allergic type-I like hypersensitivity to MC by an experiment which was designed *in vitro*.

**Methods:**

In the present study, the expression of CD63 and CD203c on peripheral blood basophils against different dilutions of MC extracts was measured using flow cytometry and compared with one another. In addition to this, intra-assay CV’s of testing extracts were calculated for precision on reproducibility of test results.

**Results:**

It was observed that the fruit extract of MC at 1/100 and 1/1000 dilutions significantly increased active basophils compared to same extract at 1/10000 dilution.

**Conclusions:**

In conclusion, *Momordica charantia* may elicit a non-allergic type-I like hypersensitivity reaction in especially susceptible individuals.

## Background

*Momordica charantia* (MC) as a member of Cucurbitaceae family has been grown in many tropical and subtropical countries and also known as bitter melon or bitter gourd [1]. This plant has traditionally been used as herbal medicine since ancient times. The therapeutic effects of this plant make it a promising candidate for development of future drugs [2]. Hence the research on drug development has been focused on investigating biochemical and pharmacological properties of this plant [3]. In this context, the numerous studies demonstrate that MC has lots of active plant chemicals such as triterpenes, proteins and steroids [4]. Accordingly, it was shown in experimental and clinical studies that each part of MC such as seed, fruit and leaf are potentially effective for treatment of various diseases such as infections, cancer, diabetes, ulcer etc [3,5,6]. Although bitter melon has not been known with its harmful side effects, the relatively low toxicity with oral intake was experienced in some *in vivo* studies [7].

The effects of MC on immune system cells were investigated by many studies *in vivo* and *in vitro* in order to explain the mechanisms of curative effects on diseases. Anti-infectious and anti-cancer effects of MC were clarified by augmentation of natural killer (NK) cell-mediated cytotoxicity and interferon-gamma production [8,9]. Bitter melon has a protein called Momordica anti-human immunovirus protein (MAP30) which is not only activates natural killer cells but also increases production of interferon gamma that fights all types of viruses. MAP30 has also capable of inhibiting infection of HIV type 1 (HIV-1) in T lymphocytes and monocytes [10]. This plant also leads to a decrease in the number of lymphocytes, increases in the populations of T helper cells and NK cells, and an increase in the immunoglobulin production of B cells [11].

It drew our attention to that although many experimental studies has been performed to reveal the effects of MC on immune functions, there is not any striking report in the literature that investigate the possible side effects of non-allergic type-I like hypersensitivity to MC and each mechanism by using immunological methods. Therefore, we decided to design an experimental study in order to show at least the effects of MC on basophils which play a great role in allergic reactions by secreting the mediators to stimulation. Briefly, the aim of this study was to investigate the response of basophils to MC plant extracts measuring the expression levels of CD63 and CD203c activation markers and to evaluate the risk of non-allergic type-I like hypersensitivity reaction to this plant. Flow cytometric analysis, a valuable and safe method for quantitative measurement of basophil activation, was used for this purpose [12].

## Methods

### Plant collection

The *Momordica charantia* L., Sp. PL.2:1009 (1753) plant was collected from the Ida mountains in August 2011. The species was characterized by Prof. Dr. Gulendam Tumen from Deparment of Biology, University of Balikesir. 60515 was given as a herbarium voucher specimen at the University of Ankara (ANK). Herbarium specimen was prepared and deposited at the Department of Biology, Faculty of Science, University of Ankara.

### Plant material extraction and preparing of dilutions

The seeds and fruits chopped from this plant were powdered by blender and dried in air. The essence of seeds and fruits of the plant were extracted separately by using hexane as solvent as described in previous report [1]. The same method was followed for extraction process of both components of bitter melon. In extraction process, each component were added to hexane in separate containers and heated overnight to obtain crude extract. After evaporation of hexane, the crude extract was dissolved in heptane and isolated from solvent by evaporation under vacuum conditions. For fruit extraction of bitter melon, a stock solution with a concentration of 161 mg/ml was prepared and this stock underwent 100 (1.61 mg/ml) to 10000 (0.0161 mg/ml) fold dilution to prepare 3 sets of samples of different concentration. For seed extraction of the plant, the same dilution procedure was applied to a concentration of 210 mg/ml to prepare 3 sets of dilutions (2.1, 0.21, 0.021 mg/ml).

### Whole blood samples

Three healthy and voluntary individuals (1 male, 2 female) were enrolled into registered for the study. Their ages were 27, 33 and 38, respectively. They did not have any acute or subacute inflammatory conditions, chronic illness including metabolic disorders or continuous drug use. Five milliliter of whole blood from each donor was drawn to anticoagulant EDTA tubes at run-time.

### Ethical approval

This study was conducted in conformity to the Helsinki Declaration and approved by the local ethics committee of Keçiören Training and Research Hospital. B.10.4. İSM.4.06.68.49/279 was given as a protocol number. All participants in my this study were volunteered and each participant agreed with their consent voluntarily).

### Basophil activation test

Basophil activation test (BAT) was carried out from whole blood including anticoagulant EDTA using flow cytometric allergen stimulation test (FlowCAST) (Bühlmann Laboratories, Schönenbuch, Switzerland) according to the manufacturer’s protocol. The staining reagents were containing a mixture of monoclonal antibodies to human CD63 and CD203c both labeled with PE-DY647, and a monoclonal antibody to CCR3 (as a selective marker for basophils) labeled with PE.

**Figure 1:**
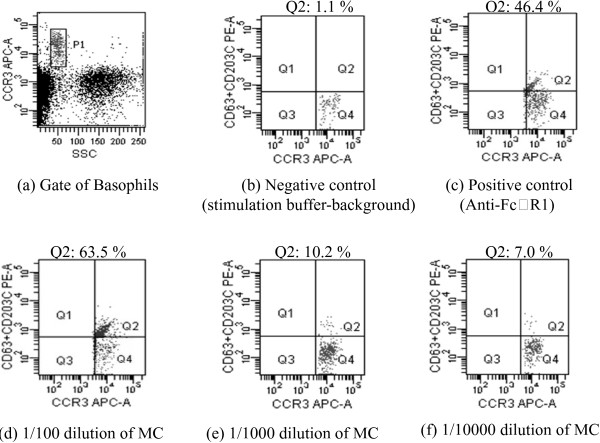
**The dot-plots from single representative experiment obtained with a healthy individual.** The basophil population was gated in an SSC-CCR3 dot plot **(a)** and analyzed for CD63 and CD203c. The percentages represent the percentage (%) of positive cells in the appropriate quadrant. The percentages of CD63 + CD203c + basophils in negative control (stimulation buffer-background) **(b)**, positive control (Anti-FcϵR1) **(c)** and whole bloods stimulated with fruit extracts of MC at 1/100 **(d)**, 1/1000 **(e)** and 1/10000 **(f)** dilutions.

Various concentrations were prepared from both seed and fruit extracts of bitter melon that were used for testing of stimulation capability of MC on basophils. For this purpose, A 100 μl of each concentrations of extracts and negative (stimulation buffer)/positive controls (anti-FcϵR1 monoclonal antibody, fMLP) were added into the sample tubes. 200 μl of stimulation buffer was mixed with samples and 100 μl of whole blood, 40 μl of staining reagent were added and then, all test tubes were incubated at 37°C for 10 min. After the incubation step, samples were allowed for eritrocyte lysing by adding 2 ml 1× lysing solution for 10 min at room temperature. Samples with lysed cells were centrifuged for 5 min at 500×g. Supernatant was discarded and pellet was resuspended with 300 μl wash buffer. Before monitoring, 800 μl wash buffer was added into each test tube. Flow cytometry was utilized for analysis of process sample (FACSCanto II, BD Bioscience, San Jose, CA using FACSDiva 5.02. software). In the low side scatter and high CCR3, positive basophil population was gated. 500 to 1000 basophil events were counted (Figure 1). Basophils were represented by expression of CD63 and CD203c (CD63 + CD203c+) were evaluated as active cells. Stimulation levels were quantified as either percentages of active basophils or mean fluorescence intensity (MFI) of dual stained basophils.

### Experimental design

Initially, a serial dilution of both fruit and seed extracts of bitter melon MC were prepared for experimental procedure as mentioned before. In the first step of the experiment, stimulation levels at three different dilutions of MC were measured in three healthy individuals, and statistically compared with one another. Thereafter, in the second step of the experiment, the measurements which were obtained from different individuals at the same dilution of MC were taken into account as though obtained same individual. Thus, the combined measurements at different dilutions of MC were statistically compared with one another.

### Intra-assay coefficients of variability

We calculated intra-assay CV’s for both controls and testing extracts in order to express the precision and reproducibility of BAT results. While the intra-assay CV’s which were calculated from the results of negative and positive controls, were in acceptable range (less than 10), testing extracts were not. We thought that some problems related to extraction procedure or solvents might lead to these unfavorable results [13-15].

Intra-assay coefficients of variability (CV) for this experimental study was separately calculated from the percentages of active basophils in 7 replicates prepared from peripheral blood samples of three healthy individuals for stimulation buffer (background-negative control), stimulation controls (anti-FcϵR1 monoclonal antibody, fMLP) and fruit extracts of MC within the same run. Results were expressed as% CV.

### Statistical analysis

All statistical analyses were performed using computer software (SPSS for Windows version 11.5, SPSS Inc., Chicago, IL, USA). For the tests of normality, we used Kolmogorov-Smirnov test. The Friedman test was used for multiple statistical comparisons. The Wilcoxon test was used as a post hoc test in order to see whether the Friedman test is statistically significant. In order to investigate the relations between the variables, we used Spearman’s rank correlation test. A p value < 0.05 was considered to be statistically significant.

## Results and discussion

Intra-assay coefficients of variabilities (CV’s) were found to be 4.2%, 5.4% and 7.6 for stimulation buffer (background-negative control) and stimulation controls (anti-FcϵR1 monoclonal antibody, fMLP), respectively. However, intra-assay CV’s calculated for different dilutions of MC (36.6%, 33.0%, 48.6, respectively) were higher than those of negative and positive controls.

No significant alteration regarding basophil activation was observed in the experiments which was done with seed extract of MC (data not shown). However, fruit extract of MC had a striking *in vitro* effect on basophils. Thus, the achieved results from this point were only related to fruit extract of MC.

**Figure 2:**
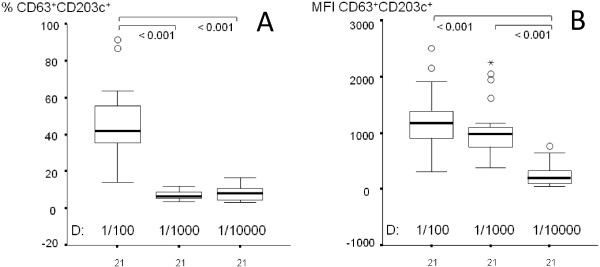
**Comparisons of stimulation levels of basophils (CD63 + CD203c+) in different dilutions of MC.** Data were obtained from combined 21 measurements of three healthy individuals, and given as percentage **(A)** or MFI **(B)**. Boxes show the ranges of 1st and 3rd quartiles and extreme values. Horizontal bars represent median values. The differences among the measurements of different dilutions were evaluated by Friedman Test. Wilcoxon Signed Rank test was used as post hoc test. p values were indicated above the boxes when a level of significance < 0.05 was reached in comparisons.

Stimulation levels at the same dilutions of MC were not different from one another in three healthy individuals (Table 1). Therefore, the combined measurements obtained from three different individuals were considered for statistical comparisons of measurements obtained from different dilutions of MC. The stimulation levels obtained from 1/100 dilution of MC were found to be higher than those of 1/1000 and 1/10000 dilutions of MC (Table 2, Figure 2). Accordingly, the percentage of CD63 + CD203c + basophils were higher at 1/100 dilution of MC than those of 1/1000 and 1/10000 dilutions of MC. However, no significant difference was found between the percentages of CD63 + CD203c + basophils at 1/1000 and 1/10000 dilutions of MC. In addition, while MFI measurements were higher at 1/100 and 1/1000 dilutions of MC than those of a 1/10000 dilution of MC, there was not any significant difference between first two dilutions. Positive correlations were found regarding to MFI measurements between 1/100 and 1/1000 dilutions of MC and between 1/1000 and 1/10000 dilutions of MC (p values: 0.029 and 0.027, respectively). However, there was not any correlation with respect to the percentages of CD63 + CD203c + basophils. These results support that fruit part of bitter melon is potentially non-allergic type-I like hypersensitivity due to stimulation of basophils *in vitro* conditions.

**Table 1:** Stimulation levels of basophils in three healthy individuals

**Individuals**	**1/100**		**1/1000**		**1/10000**	
	**%**	**MFI**	**%**	**MFI**	**%**	**MFI**
1	37.7(49.4)	1414(1381)	5.6(6.2)	1112(1857)	6.9(10.7)	234(595)
2	37.0(28.5)	882(1078)	6.0(8.1)	654(703)	9.0(8.3)	152(123)
3	57.0(57.0)	1238(1639)	8.2(6.1)	932(877)	9.9(13.7)	345(698)

Data were expressed as median (range).

**Table 2:** Stimulation levels of basophils in combined measurements

**1/100**		**1/1000**		**1/10000**	
**%**	**MFI**	**%**	**MFI**	**%**	**MFI**
42.1(79.9)	1187(2227)	6.2(8.3)	988(1892)	8.0(13.7)	212(728)

Data were expressed as median (range).

In spite of discouragement in intra-assay CV’s from different dilutions of MC, significant differences with respect to both percentages and MFI values obtained from BAT were seen in statistical comparisons of different dilutions of MC extracts prepared from fruit part. In addition, positive correlations between different dilutions of MC extracts were found regarding MFI measurements. However, no significant difference in the same respects was found among the comparisons of different dilutions of MC extracts prepared from seed part. These results may be explained with the effects of various constituents available in different parts of bitter melon such as fruit, seed, leaf and root [8]. The principal constituents are alpha and beta-momorcharin in fruits, seeds and leaves; insulin-like polypeptides (p-insulin, alkaloid momordicine) in both the fruits and seeds; steroid glycosides (momordin, charantin) in both fruits and leaves; vicine and stearic-, linoleic-, and oleaic-acids in seeds; iron, sodium, thiamine, riboflavin, niacin, and ascorbic acid in leaves [16,17]. So indeed, all parts of bitter melon have common beneficial and deleterious impacts such as anti-infectious, anti-inflammatory, anti-cancer, anti-diabetic, anti-ulcer, anti-fertility and low toxicity.

Despite the lack of detailed research on non-allergic type-I like hypersensitivity effect of bitter melon, a few reports that mention allergic properties of Cucurbitaceae family exist in the literature [18-24]. Satar and Kloutsos et al. reported cases which presented life-threatening uvular angioedema and upper airway edema resulting from using of Ecballium elaterium known as the wild cucumber which is a plant indigenous to the Mediterranean region [20,21]. Zucchini (Cucurbita pepo) which is also a member of the Cucurbitaceae family, was studied by Reindl et al. In the study of Reindl et al, allergic reaction to this plant in 4 patients with oral allergy syndrome, nausea, diarrhea, or pruritus were identified after the intake of zucchini [22].

## Conclusion

In conclusion, *Momordica charantia* or Bitter Melon, is a tropical plant, has been consumed extensively in folk medicine. The parts of this plant have similar or different effects that are not only beneficial, but also harmful. There are many handbook and website sources including knowledge about bitter melon. However, further in vivo and in vitro studies are needed in order to reveal many more unknown properties of this plant including both therapeutic and adverse effects.

## Abbreviations

MC: *Momordica charantia*; MFI: Mean fluorescent intensity; BAT: Basophil activation test.

## Competing interests

The author declares that he has no competing interests.

## Authors’ contribution

I have designed this study and have conducted all experiments, acquisition and analyses of all flow cytometric data. I also drafted manuscript.

## Pre-publication history

The pre-publication history for this paper can be accessed here:

http://www.biomedcentral.com/1472-6882/13/284/prepub
